# Aural Furunculosis

**Published:** 1909-07-10

**Authors:** 


### Otology.

### AURAL FURUNCULOSIS.

Furuncles, or boils, are caused by the entrance of micro-organisms, especially the various kinds of staphylococci, into the sebaceous glands of the skin, and it is not surprising that they occur fairly frequently in the external auditory meatus where micro-organisms have a chance of remaining and multiplying more or less undisturbed. Furunculosis may be readily produced artificially by rubbing staphylococci into the skin, and shows a natural tendency to occur on parts which are subject to friction; it is therefore probable that the exciting cause of aural furuncles is usually some slight trauma, such as scratching with a hair-pin, or the bad habit of attempting to clean the meatus by the forcible insertion of a roll of towel. A slight eczema or superficial dermatitis also frequently precedes the attack of boils. They are commoner in women, especially anæmic delicate women, than in men, and are rarely observed in young children.

Though a minor affection, aural furunculosis is an extremely painful one. The pain radiates over the corresponding half of the head, is increased by mastication, and, like other forms of inflammatory pain, is worse at night. There is considerable fever and general constitutional disturbance, combined with sleeplessness and depression. When the external meatus is blocked by swelling there is some impairment of hearing, and possibly this is increased by the accompanying injection of the membrana tympani, but the deafness is not very marked, a useful point of distinction between this affection and acute otitis media.

On examination the meatus is seen to be swollen. The degree of swelling is very variable; it may be confined to the neighbourhood of a single boil, or it may involve the entire meatus and the tragus. In extreme cases there is considerable redness and swelling over the mastoid region causing the auricle to project, but when this is the case the swelling always fills up the groove between the back of the concha and the head. The introduction of a speculum is extremely painful, and the utmost gentleness is required. At an early stage there may be no localisation of the inflammation, and the condition may be called "diffuse external otitis"; or the attack may begin as a circumscribed boil and the inflammation may subsequently spread and become diffuse. Thus there is no sharp line of demarcation between furunculosis or circumscribed external otitis and the diffuse form. The boils occur in the cartilaginous meatus, and it is the close connection between the skin and perichondrium which renders the affection so acutely painful. They appear first as red elevations, and if suppuration occurs yellow spots become visible, which discharge pus; a necrosed centre is extruded before healing can occur. Sometimes small granulations sprout through the opening; on the other hand, a boil may subside without proceeding to suppuration.

The diagnosis is generally a simple matter, but it must be remembered that acute otitis media is sometimes accompanied by great swelling of the meatus, and it therefore becomes of great importance to exclude the latter affection in any case apparently one of furunculosis. The pain, fever, and constitutional disturbance may be as great in the one as in the other, and in the early stage of otitis the discharge may be no more than is often seen in furunculosis. The diagnosis is clear if a definite furuncle can be seen. The bulging of the postero-superior meatal wall, which occurs in acute mastoiditis, must not be mistaken for a boil; the former is flatter and less prominent, and is more deeply situated within the osseous part of the canal. In furunculosis, if the ear is carefully and gently cleaned with cotton-wool mops, the membrane can frequently be seen; though often reddened superficially, it is neither deeply congested nor bulging. The hearing also is much less impaired than in a case of acute otitis media. When there is marked redness and swelling over the mastoid region there is a decided superficial resemblance to acute inflammation within the mastoid process; but there is no pain on deep pressure over the mastoid, though there may be superficial tenderness, and the swelling always occupies the retro-auricular groove, which is never obliterated by true mastoid disease.

To relieve the pain hot instillations are most valuable, such as Cocainæ hydrochlor. gr. x., morphinæ hydrochlor. gr v., aquam ad ℥j., and they should be employed as hot as can be borne. Fomentations should not be used, as they increase the congestion and favour the growth of micro-organisms. The application of cold, though sometimes giving relief, is not to be recommended as a rule. The boils should be opened as soon as they appear, without waiting for the yellow point of suppuration; a special angular furuncle knife is advisable. Nitrous oxide anæsthesia is most necessary, as the operation is extremely painful, and local anæsthetics are useless in this situation. Careful antisepsis is of great importance; the meatus should be syringed with a fairly strong antiseptic, such as 1-1000 perchloride of mercury, and all desquamated epithelium and other debris must be carefully removed. Instillations of rectified spirit containing boracic acid, gr. xv. to the ounce, may then be used three or more times a day. When pain is severe the application of leeches in front of the tragus will give much relief, but leaves rather conspicuous scars. Of general treatment, a brisk purge is indicated at the outset, and calcium sulphide, gr. $\frac{1}{4}$ to 1, is often prescribed. Tonics are, however, more to be recommended, iron and arsenic and, especially, dilute sulphuric acid in rather large doses of twenty to thirty minims three times a day. Those who are subject to attacks of furunculosis should have the ears cleared of cerumen at frequent intervals, and may with advantage massage the meatus with a probe armed with cotton-wool anointed with a weak mercurial ointment consisting of 1 drachm of unguentum hydrargyri nitratis to an ounce of vaseline.

